# Microscopic Polyangiitis With Non-fibrotic Hypersensitivity Pneumonitis Patterns on Chest Computed Tomography: A Case Report

**DOI:** 10.7759/cureus.93148

**Published:** 2025-09-24

**Authors:** Kodai Ueda, Masashi Matsuyama, Eri Takeuchi, Shu Teshima, Mizu Nonaka, Yuko Minami, Takefumi Saito, Yukio Ishii, Nobuyuki Hizawa

**Affiliations:** 1 Department of Pulmonary Medicine, National Hospital Organization Ibarakihigashi National Hospital, Tokai, JPN; 2 Department of Pulmonary Medicine, University of Tsukuba, Tsukuba, JPN; 3 Department of Pathology, National Hospital Organization Ibarakihigashi National Hospital, Tokai, JPN

**Keywords:** anti-neutrophil cytoplasmic antibody-associated vasculitis, high-resolution computed tomography, interstitial lung disease, microscopic polyangiitis, non-fibrotic hypersensitivity pneumonitis

## Abstract

In microscopic polyangiitis (MPA)-associated interstitial lung disease (ILD), the usual interstitial pneumonia (UIP) is the most common pattern. We report a rare case of a 71-year-old woman with MPA initially suspected to have non-fibrotic hypersensitivity pneumonitis (HP) based on chest computed tomography (CT) findings of diffuse ground-glass opacities, mosaic attenuation, and air trapping. HP was excluded due to the lack of exposure to inhaled antigens, lack of response to inpatient antigen isolation, and absence of lymphocytosis in bronchoalveolar lavage fluid. The diagnosis of MPA was established based on the presence of ILD and renal impairment in combination with elevated myeloperoxidase-antineutrophil cytoplasmic antibody levels. This case highlights the rarity of a non-fibrotic HP pattern in MPA and the importance of integrating clinical, radiological, and serological data in evaluating MPA with atypical chest CT imaging.

## Introduction

In microscopic polyangiitis (MPA), alveolar hemorrhage and interstitial lung disease (ILD) are the major pulmonary manifestations [1]. ILD occurs in approximately half of all patients with MPA [2]. Common computed tomography (CT) findings in MPA include ground-glass opacities (GGOs), reticular patterns, traction bronchiectasis, interlobular septal thickening, bronchial wall thickening, consolidations, and honeycombing [2,3]. These findings are diverse and may complicate the differential diagnosis.

Usual interstitial pneumonia (UIP) pattern is the most common CT imaging pattern of MPA-associated ILD (MPA-ILD) [2,4]. Other reported imaging patterns include nonspecific interstitial pneumonia (NSIP) and desquamative interstitial pneumonia (DIP), but reports of additional rare imaging patterns are limited [4]. In particular, cases of MPA presenting with a non-fibrotic hypersensitivity pneumonitis (HP) pattern have rarely been reported. Such atypical CT findings may hinder the diagnosis of MPA.

HP is an immune-mediated disease caused by exposure to inhaled antigens and typically presents with imaging findings, such as GGOs, centrilobular nodules, mosaic attenuation pattern, and air trapping [5]. Since some of these findings overlap with MPA-ILD, differential diagnosis using clinical and laboratory findings is important.

In this report, a case of MPA with a non-fibrotic HP pattern on chest CT is described. Through careful history-taking and evaluation of clinical and laboratory findings that were inconsistent with HP, the diagnosis of MPA was made.

## Case presentation

A 71-year-old Japanese woman was admitted to our hospital with fever, cough, and exertional dyspnea. She had hypertension and hyperlipidemia, for which she was undergoing long-term treatment with azilsartan (20 mg) and ezetimibe (10 mg). She had no history of smoking or allergies. She had previously worked in an office-based occupation. Environmental history was carefully assessed using a questionnaire, revealing no contact with birds and no evidence of mold exposure in the home, nor any other inhaled antigens commonly associated with HP. Prior to admission, she had been prescribed antibiotics at a previous clinic without improvement.

On admission, her physical findings were as follows: height of 145.1 cm, weight of 46.4 kg, temperature of 38.1°C, blood pressure of 121/79 mmHg, heart rate of 93 bpm, respiratory rate of 16 breaths per minute, and SpO_2_ of 97% on room air. Breath sounds were clear with no abnormalities.

Laboratory findings on admission are shown in Table 1. Blood tests showed leukocytosis with neutrophilic predominance, anemia, thrombocytosis, elevated hepatobiliary enzymes, increased C-reactive protein (CRP; 22.35 mg/dL), and hypoalbuminemia. Krebs von den Lungen-6 (KL-6) was not elevated.

**Table 1:** Laboratory test results on admission

Parameter	Result	Normal Range
White blood cell count (/μL)	13,500	3500-9000
Neutrophils (%)	82	45-70
Lymphocytes (%)	11	20-40
Monocytes (%)	6	1-9
Eosinophils (%)	1	0-5
Hemoglobin (g/dL)	9.8	12.0-15.0
Platelet count (×10^3^ /μL)	554	150-400
Albumin (g/dL)	2.0	3.8-5.3
Aspartate aminotransferase (U/L)	90	13-33
Alanine aminotransferase (U/L)	68	6-27
Lactate dehydrogenase (U/L)	193	119-229
Blood urea nitrogen (mg/dL)	26.8	8-22
Creatinine (mg/dL)	0.94	0.40-0.70
C-reactive protein (mg/dL)	22.35	0-0.30
Krebs von den lungen-6 (U/mL)	333	0-500

**Figure 1:**
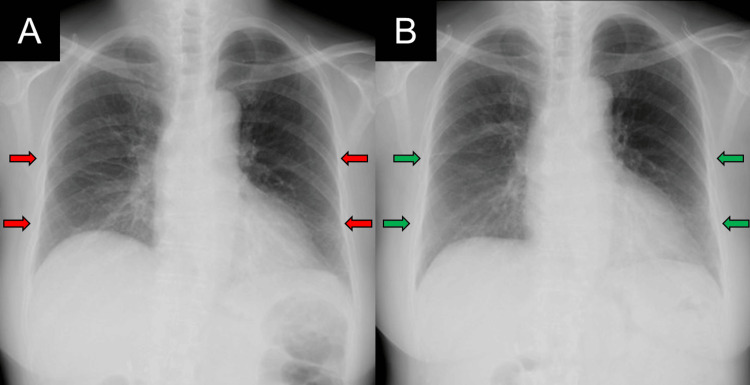
Chest radiographs on admission (A) and at two months after combination therapy (B) (A) Chest radiograph on admission showing bilateral diffuse ground-glass opacities (GGOs, red arrows). (B) Chest radiograph at two months after combination therapy showing improvement in lung volumes and GGOs (green arrows).

**Figure 2:**
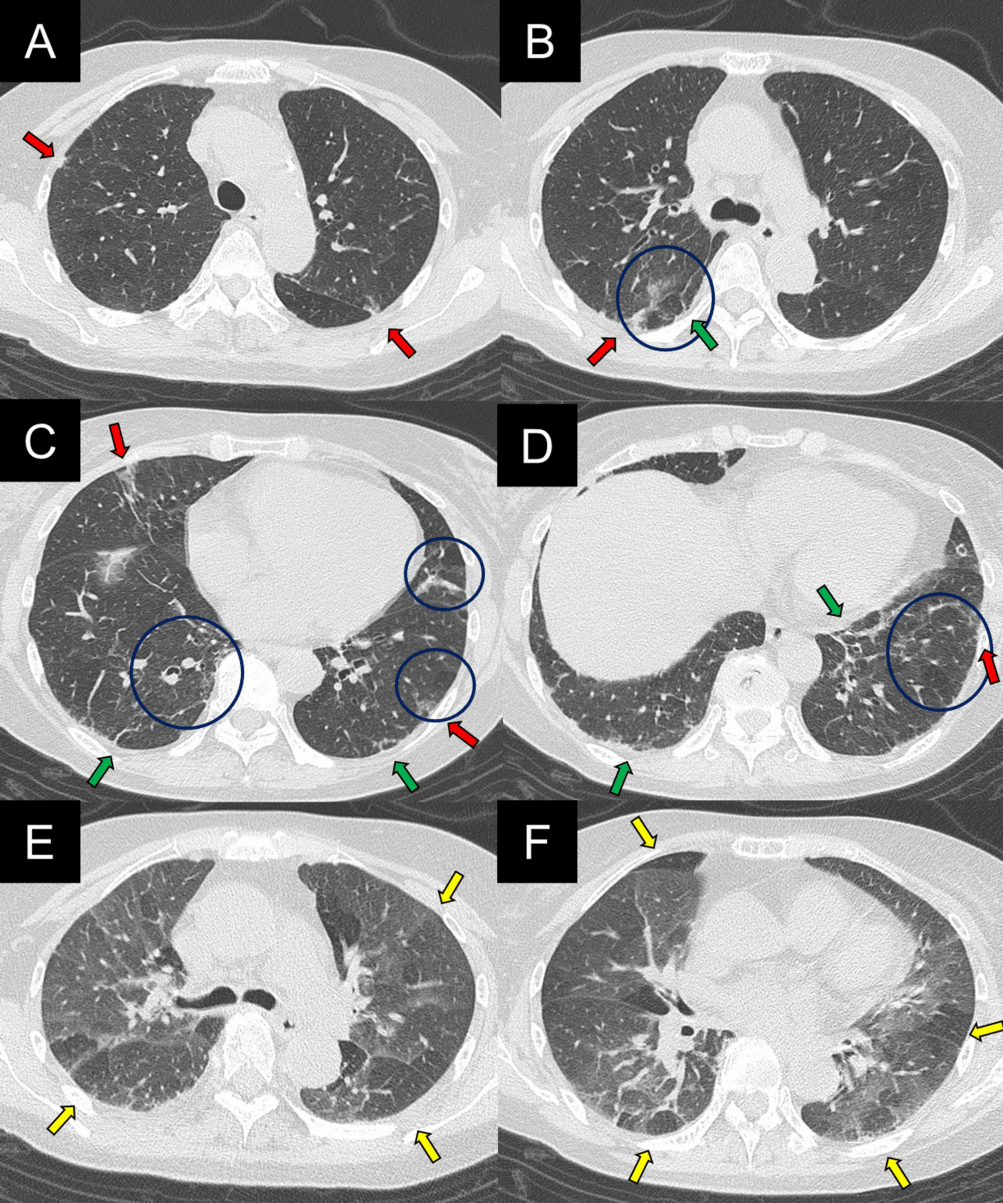
High-resolution computed tomography (HRCT) on admission (A-D) Inspiratory phase images showing diffuse ground-glass opacities (GGOs, red arrows), mosaic attenuation (blue circles), and linear opacities (green arrows). (E-F) Expiratory phase images showing air trapping (yellow arrows).

Imaging findings on admission are shown in Figures 1A, 2. The chest radiograph showed reduced lung volumes and bilateral diffuse GGOs. High-resolution chest CT (HRCT) demonstrated diffuse GGOs, mosaic attenuation, and linear opacities. These findings were diffusely distributed in both craniocaudal and axial directions. Expiratory HRCT demonstrated prominent air trapping. These HRCT findings were considered typical for a non-fibrotic HP pattern according to the American Thoracic Society (ATS) guideline [5]. Abdominal and sinus CT scans showed no abnormalities.

Based on the imaging findings, non-fibrotic HP was considered, and inpatient antigen isolation was started as a diagnostic trial from the first to fifth hospital day. However, her clinical findings did not improve.

**Figure 3:**
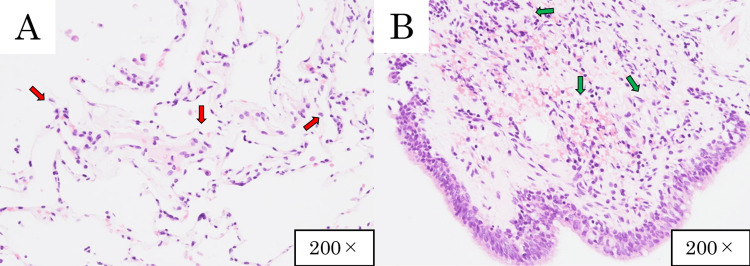
Histopathological image of the transbronchial lung biopsy with hematoxylin-eosin stain (×200) (A) The lung interstitium shows mild lymphocytic infiltration (red arrows), with no evidence of fibrosis, granuloma formation, or intra-alveolar macrophage accumulation. (B) The bronchiolar interstitium shows lymphocytic infiltration (green arrows).

Further investigations were conducted, as shown in Tables 2-4. Immunoserological testing showed a marked increase in the myeloperoxidase-antineutrophil cytoplasmic antibody (MPO-ANCA) level to more than 134 U/mL. In addition, urinalysis showed urinary occult blood and urinary protein. Information obtained from the previous physician showed that the patient’s serum creatinine level had increased from a prior value of 0.60-0.94 mg/dL at the time of admission. Bronchoalveolar lavage fluid (BALF) showed no evidence of alveolar hemorrhage and no increase in lymphocytes. In non-fibrotic HP, the lymphocyte fraction in BALF is typically >20-30% [5], but in this case, it was only 2%. Transbronchial lung biopsy (TBLB) findings showed lymphocytic infiltration in the bronchial walls (Figure 3). Histopathological findings characteristic of HP, such as intra-alveolar macrophages, non-caseating granulomas, and fibrosis [5], were not observed. Based on the CT findings (Figure 2), markedly elevated serum MPO-ANCA levels (>134 U/mL), along with urinary protein, occult blood, and renal dysfunction, the patient was diagnosed with MPA. According to the 2022 American College of Rheumatology (ACR)/European Alliance of Associations for Rheumatology (EULAR) classification criteria for MPA, the patient’s score was nine, exceeding the threshold of five points required for classification [6]. HP was considered low confidence according to the ATS guideline [5], given the lack of antigen exposure, lack of response to antigen avoidance, and absence of lymphocytosis in BALF. It was decided not to perform a renal biopsy to expedite treatment for MPA.

**Table 2:** Results of investigations into the cause of interstitial lung disease CCP, cyclic citrullinated peptide; AB, antibody; SS, Sjögren's syndrome; ARS, aminoacyl-tRNA synthetase; PR3-ANCA, proteinase 3 anti-neutrophil cytoplasmic antibody; MPO-ANCA, myeloperoxidase-antineutrophil cytoplasmic antibody; IG, immunoglobulin

Parameter	Results	Normal range
Anti-CCP AB	(-)	(-)
Anti-nuclear AB	1:80	<1:40
Anti-SS-A AB (U/mL)	38.9	0-9.9
Anti-SS-B AB	(-)	(-)
Anti-topoisomerase I antibody	(-)	(-)
Anti-ARS antibody	(-)	(-)
PR3-ANCA	(-)	(-)
MPO-ANCA (U/mL)	>134	0-3.4
Specific IgG antibody to bird AB	(-)	(-)
Anti-*Trichosporon asahii* AB	(-)	(-)
Urine protein	(2+)	(-)
Urine occult blood	(2+)	(-)

**Table 3:** Results of pulmonary function tests

Parameter	Results	% of predicted
Total lung capacity (L)	2.40	68.0%
Residual volume (L)	1.18	85.5%
Forced vital capacity (L)	1.26	61.8%
Forced expiratory volume in one second (L)	1.07	66.0%
Forced expiratory volume % in one second(%)	84.92	106.8%
DL_CO_ (mL/min/mmHg)	8.71	67.4%

**Table 4:** Results of bronchoalveolar lavage analysis

Parameter	Results	Normal range
Recovery volume	86/150 mL	55-105/150 mL
Appearance	Slightly turbid white	Turbid white to clear
Hemosiderin-laden macrophages	(-)	(-)
Total cells (/μL)	130	70-200
Macrophages (%)	97	>85
Lymphocytes (%)	2	10-15
Neutrophils (%)	1	<3
Eosinophils (%)	0	<1
CD4/CD8	0.9	0.9-2.5
Culture	Normal flora	Normal flora

Although anti-SS-A antibodies were elevated (Table 2), the patient exhibited no clinical signs of Sjögren’s syndrome, such as dry mouth or dry eyes. Head CT showed no abnormalities in the salivary glands, and ophthalmological examination showed no findings suggestive of Sjögren’s syndrome. Serological tests did not indicate the presence of other connective tissue diseases associated with ILD (Table 2).

**Figure 4:**
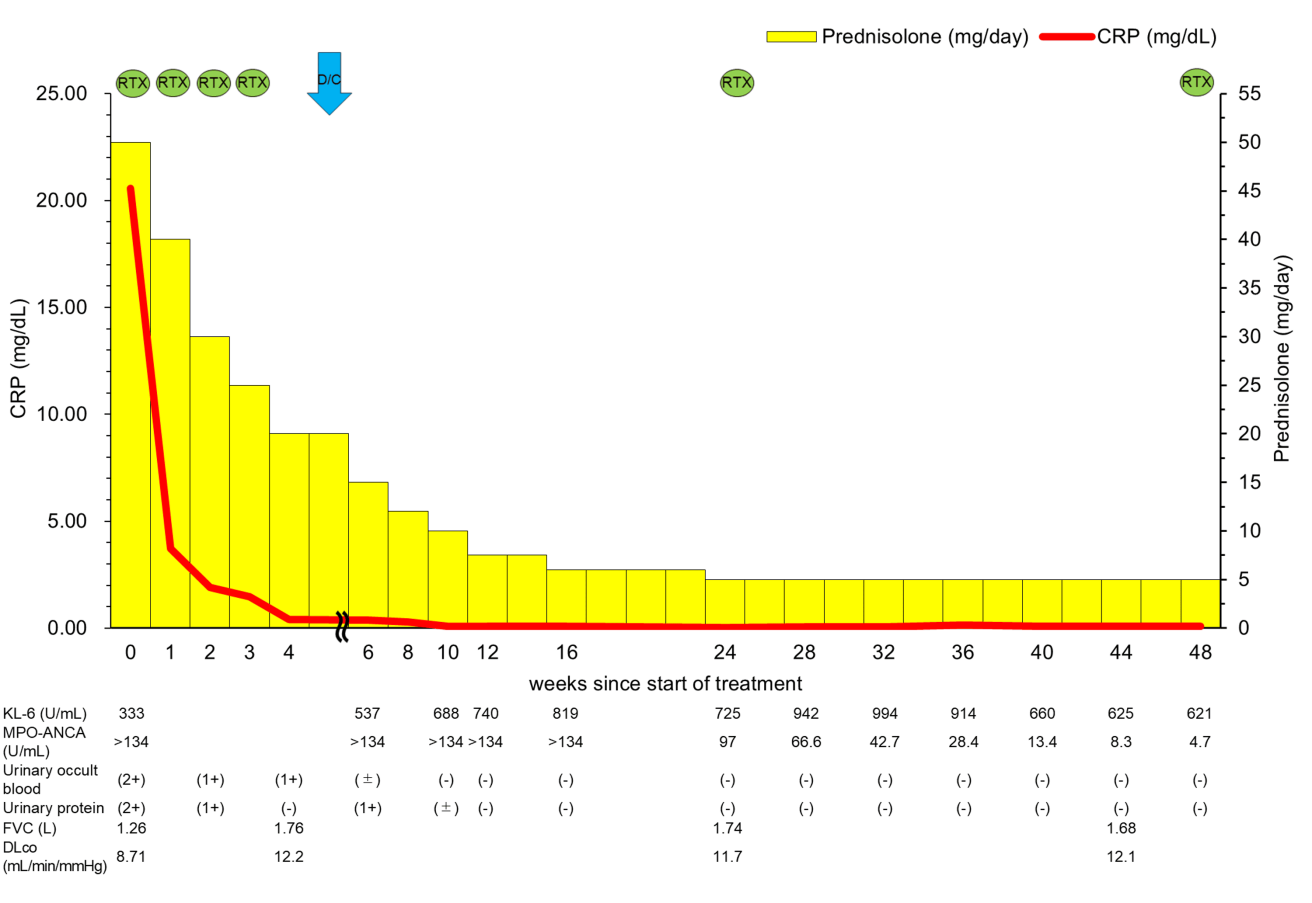
Overview of the patient’s clinical course The patient’s clinical course. The laboratory data and treatment details are shown. CRP, C-reactive protein; D/C, discharge; FVC, forced vital capacity; KL-6, Krebs von den lungen-6; MPO-ANCA, myeloperoxidase-antineutrophil cytoplasmic antibody; RTX, rituximab

The clinical course is summarized in Figure 4. On the fifth hospital day, treatment with prednisolone was started at 50 mg/day (1 mg/kg/day). From the ninth hospital day, rituximab was administered at 500 mg/body (375 mg/m^2^) once weekly for four cycles.

**Figure 5:**
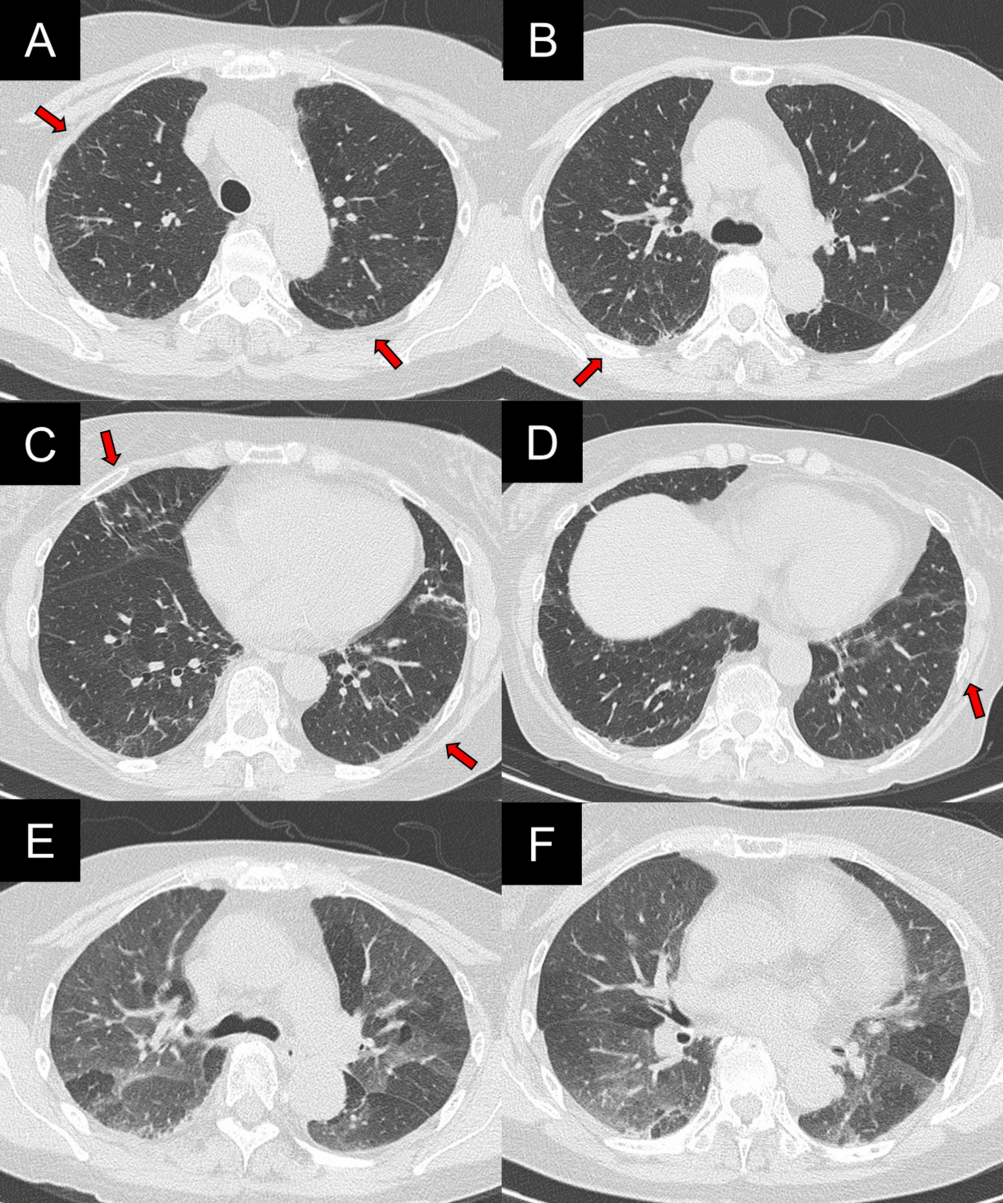
High-resolution computed tomography (HRCT) obtained two months after combination therapy (A-D) Inspiratory phase images showing improvement of GGOs (red arrows). (E-F) Expiratory phase images.

The patient’s symptoms improved significantly with treatment. CRP levels normalized, and urinary occult blood and proteinuria improved (Figure 4). The chest radiograph (Figure 1B) and chest HRCT (Figure 5) showed improvement in lung volumes and GGOs. Pulmonary function tests (PFT) showed improvement: forced vital capacity increased from 1.26 to 1.76 L, and diffusing capacity for carbon monoxide also increased from 8.72 to 12.2 mL/min/mmHg (Figure 4). Prednisolone was tapered gradually, and by the 37th hospital day, the dose was reduced to 20 mg/day. The patient was discharged on the 40th hospital day.

In outpatient follow-up (Figure 4), CRP and urinalysis findings remained stable. Prednisolone was further tapered to 5 mg/day five months after discharge. Although KL-6 levels increased transiently during outpatient monitoring, they subsequently decreased. MPO-ANCA levels also decreased from >134 to 4.7 U/mL (Figure 4). Prednisolone was to be further tapered in follow-up. Rituximab was scheduled to be administered every six months for a total of 18 months after discharge.

## Discussion

This case highlights two key points: first, MPA may show imaging findings of a non-fibrotic HP pattern; and second, diagnosis requires integration of the clinical course and laboratory data, in addition to imaging.

First, MPA may show imaging findings of a non-fibrotic HP pattern. According to the ATS guideline [5], the typical HRCT pattern for non-fibrotic HP includes parenchymal infiltration, such as GGOs and mosaic attenuation, as well as abnormalities indicative of small airway disease, such as air trapping and centrilobular nodules. The distribution of parenchymal abnormalities is diffuse in both the craniocaudal and axial planes. Although these findings are characteristic of non-fibrotic HP, they are nonspecific. Therefore, identification of antigen exposure, along with BALF and histopathological findings, is important for an accurate diagnosis [5]. In the present case, chest HRCT showed diffuse GGOs, a mosaic attenuation pattern, and air trapping. The distribution of lesions was diffuse in both the craniocaudal and axial directions. These findings were the typical imaging pattern of non-fibrotic HP [5].

Though HP patterns have been rarely reported in cases of ANCA-associated vasculitis (AAV)-ILD [7,8], to the best of our knowledge, no case reports have described MPA presenting with a non-fibrotic HP pattern. One possible reason for this is that guidelines for HP imaging have been established and clarified only in the past few years [5]. There are only a limited number of articles describing a direct association between MPA and HP, and there was one case report of MPA combined with fibrotic HP [9], but it was fibrotic HP, different from the present case.

UIP pattern is the most common CT pattern of MPA [2,4], and other patterns such as NSIP and DIP have also been described [4]. However, only a few cases have been reported to show CT patterns corresponding to specific idiopathic interstitial pneumonias [4]. In MPA-ILD, atypical features for UIP pattern, such as GGOs, consolidations, and bronchial wall thickening, have been reported [2-4]. Moreover, a mosaic attenuation pattern, which is one of the characteristics of non-fibrotic HP, has been reported in 7.0-9.0% of MPA cases [2,3].

From a pathological perspective, though UIP is common in AAV-ILD, previous studies have reported features distinguishing AAV-ILD from idiopathic pulmonary fibrosis, such as bronchiolitis and follicular lymphoid hyperplasia [10]. It is possible that these pathological findings were recognized as imaging findings of a non-fibrotic HP pattern. In non-fibrotic HP, lymphocyte-predominant cellular bronchiolitis is commonly observed [5]. In the present case, the histological findings showed lymphocytic infiltration in the bronchial walls, a feature that can be seen in both MPA and non-fibrotic HP, thus making the histopathological distinction challenging in the present case. When encountering ILD with involvement of the small airways, AAV-ILD should be considered in the differential diagnosis.

Some studies have suggested that environmental factors such as silica exposure may contribute to the development of AAV [11]. This raises the possibility that antigen exposure could be a common feature linking MPA and HP. However, in the present case, no specific inhaled antigen was identified.

Second, this case emphasizes the importance of incorporating not only imaging findings, but also the clinical course and laboratory data in differentiating MPA and HP. On admission, the diagnostic approach was initially guided by chest CT findings suggestive of non-fibrotic HP. In addition, symptoms such as fever, cough, and exertional dyspnea were considered to be consistent with HP. However, the patient did not respond to inpatient antigen isolation, and BALF showed no lymphocyte elevation, with a lymphocyte fraction of 2%, a finding atypical for non-fibrotic HP [5]. Alveolar hemorrhage was also absent in the BALF. Furthermore, no obvious exposure to inhaled antigens was identified in the clinical history, and MPO-ANCA levels were markedly elevated to ≥134 U/mL. Urinalysis showed urinary occult blood and urinary protein. Based on these findings, non-fibrotic HP was considered unlikely. When chest imaging suggests HP, but no causative antigen exposure is identified, and abnormal laboratory findings such as urinary occult blood or proteinuria are present, other diseases that can cause ILD, including MPA, should be considered in the differential diagnosis.

In this case, after initiation of treatment, the patient’s symptoms resolved, and PFT improved to normal levels. In AAV-ILD, the UIP pattern is known to be associated with a worse prognosis than non-UIP patterns [12,13]. Therefore, the outcome in this non-fibrotic HP pattern may be more favorable compared with that of the UIP pattern.

Although there are reports that indirectly support the diagnostic utility of inpatient antigen isolation in HP, the duration of isolation varies across studies [14]. No studies have systematically evaluated the diagnostic accuracy of patient responses to antigen avoidance [14]. Therefore, the usefulness of antigen isolation in the present case should be interpreted with these limitations in mind.

The diagnosis of MPA was made using the 2022 ACR/EULAR classification criteria for MPA [6]. The patient lacked characteristics of granulomatosis with polyangiitis or eosinophilic granulomatosis with polyangiitis, but had an elevated MPO-ANCA titer and the presence of ILD, leading to a diagnosis of MPA based on the above criteria [6]. It is important to recognize that MPO-ANCA is not specific for MPA: false-positive results have been reported in other diseases and vasculitides, while some patients with MPA may test negative [15]. Urinalysis results suggesting renal impairment due to MPA and the presence of high CRP levels (22.35 mg/dL) at the time of hospitalization may have been clues to the diagnosis of MPA. This case underscores the need to evaluate a broad spectrum of differential diagnoses when assessing ILD.

Treatment was conducted in accordance with the ACR guideline for MPA [16] and resulted in significant clinical improvement. Though multiple treatment studies for MPA itself have been conducted, there is limited evidence regarding the management of AAV-ILD [12]. Combination therapy with corticosteroids and immunosuppressants has been reported as a good approach, as well as MPA, in a few studies [4,17]. Although AAV-ILD has been linked to a higher risk of mortality in the presence of lung function decline, UIP pattern, or elevated MPO-ANCA [13], our patient achieved a favorable outcome with early therapeutic intervention.

## Conclusions

A rare case of MPA initially suspected to be non-fibrotic HP based on chest CT findings was described. In conclusion, MPA-ILD may occasionally mimic the radiological pattern of non-fibrotic HP. In this case, however, the absence of environmental antigen exposure, lack of lymphocytosis in BALF, elevated MPO-ANCA levels, and abnormal urinary findings were atypical for HP and instead supported the diagnosis of MPA. Therefore, a comprehensive assessment, including clinical history and laboratory results, is crucial for accurate diagnosis.
